# Psychometric Properties of the Scale for Subjective Somatic and Cognitive Complaints of Psychotropic Medication Adult‐Aged‐Spectrum (SCOPA)

**DOI:** 10.1002/hup.70009

**Published:** 2025-07-09

**Authors:** Levi J. M. Schuurman, Sjacko Sobczak, Julie E. M. Schulkens, Benno P. F. Balter, Sebastiaan P. J. van Alphen

**Affiliations:** ^1^ Department of Neuropsychology and Psychopharmacology Faculty of Psychology and Neuroscience (FPN) Maastricht University Maastricht the Netherlands; ^2^ Clinical Center of Excellence for Older Adults with Personality Disorders Mondriaan Mental Health Center Heerlen Maastricht the Netherlands; ^3^ Rotterdam University of Applied Sciences Rotterdam the Netherlands; ^4^ Department of Psychiatry and Neuropsychology Faculty of Health and Life Sciences (FHML) School for Mental Health and Neuroscience (MHeNs) Maastricht University Maastricht the Netherlands; ^5^ Vrije Universiteit Brussel (VUB) Personality and Psychopathology Research Group (PEPS) Brussels Belgium

**Keywords:** construct validity, internal consistency, older adults, pharmacotherapy, psychotropic medication, side effects

## Abstract

**Objective:**

Patient‐reported side‐effect questionnaires contribute to balanced decisions regarding treatment strategy among patients with mental health disorders. However, current side‐effect questionnaires are less suitable for older adults because they often lack age‐specific side effects. Therefore, the Scale for Subjective Somatic and Cognitive Complaints of Psychotropic Medication Adult‐Aged‐Spectrum (SCOPA) was developed to quantify psychotropic side effects across the full adult age spectrum. The aim of this study was to develop and evaluate the reliability and validity of the SCOPA.

**Procedures:**

Psychometric properties of the SCOPA were assessed in a Dutch patient population (*n* = 205, age 18–86 years) and a general population (*n* = 281, age 18–87 years). Reliability of the subscales was measured through average inter‐item correlation and Cronbach's alphas. Construct validity was analysed by exploratory factor analysis.

**Results:**

Average inter‐item correlation and Cronbach's alphas of the subscales of the SCOPA were moderate to good. Exploratory factor analyses supported a two‐factor solution, labelled as complaints in somatic and cognitive domains.

**Conclusions:**

This study showed that the SCOPA has promising psychometric qualities and practical capability to evaluate the side‐effect burden of treatment with psychotropic medication across the adult‐aged‐spectrum. However, more research is needed to cross‐validate these results in the field's pursuit of increased patient safety.

## 1 Introduction

The general increased life expectancy worldwide is a fact and the number of individuals facing mental health disorders is rising (Collishaw 2014; McManus et al. 2019; Santomauro et al. 2021; Ford et al. 2019). The prevalence of mental disorders had already increased significantly by 2.47% between 1990 and 2017. Years lived with disability due to mental illness have risen by 13.5% over the past decade (Richter et al. 2019). The prevalence of mental health disorders is especially high among older adults (> 65 years); almost 20% of older adults seen in primary care have mental problems (Abdoli et al. 2021; McCombe et al. 2018). In 2016, 25% of older adults 65 years and older and living at home, as well as 74% of those living in institutions, received psychotropic medication for their mental illnesses (Loggia et al. 2020). Moreover, the number of psychotropic medications (ranging from one up until five each day) represents more than 10% of total daily medication intake (Arnold et al. 2017; Harrison et al. 2018; Varma et al. 2010). Psychotropic medications can cause a range of severe physical complaints and complications, like an increased risk of falling accidents and hospitalisations (Scott and Jayathissa 2009). Pharmacotherapy is especially complex in older adults for multiple reasons. For example, there is high comorbidity of mental, cognitive, and somatic disorders (multimorbidity), which may interact with pharmacological treatment, worsening daily life functioning, medication compliance, and well‐being (Dols et al. 2014; McCloughen et al. 2012; Skoog 2011; Van Der Wolf et al. 2017). Moreover, polypharmacy is highly prevalent in older adults, which causes interactions between medications (Maher et al. 2013). Plus, certain classes of psychotropic medications have been shown to increase the risk of obesity and diabetes (Consensus Development Conference on Antipsychotic Drugs and Obesity and Diabetes et al. 2004). This is especially alarming, with cardiovascular risk factors already more prevalent in older adults (Rodgers et al. 2019). Finally, as a result of changing pharmacodynamics, side effects tend to be 3 times more prevalent above the age of 50 years (Lingjærde et al. 1987; Turnheim 2003). Due to the above reasons, different complications (e.g., falls and hospital admissions) and side effects (e.g., in sexual and cognitive functioning) can occur in older adults more often (Gustafsson et al. 2016; Serretti and Chiesa 2010). More frequent side effects have been associated with lower quality of life, non‐adherence to therapy, and reduced treatment efficacy (Olsson et al. 2011; Semahegn et al. 2018). Hence, in clinical practice, the balance between treating symptoms adequately and side‐effect impact needs careful consideration. One way that contributes to balanced decisions is identifying the prevalence and impact of side effects through the use of patient‐reported side‐effect questionnaires.

A self‐report tool is especially important when the measured construct is foremost a subjective experience, like with side effects. Moreover, self‐report questionnaires can facilitate reports of side effects that are perceived as too sensitive to disclose. Patient‐reported questionnaires provide extra information compared to clinician‐rated questionnaires and are of value in treatment and disease management and mental healthcare (Anderson et al. 2011; Lindström et al. 2001; Nakonezny et al. 2010; Pandina et al. 2010). For instance, commonly used side‐effect questionnaires, including the UKU Side Effect Rating Scale, self‐rating version (UKU‐SERS‐Pat), Glasgow Antipsychotic Side‐effect Scale (GASS), and the Liverpool University Neuroleptic Side Effect Rating Scale (LUNSERS), have established differences in the severity of side effects across sexes and medication doses and have identified questionnaire items predictive of antipsychotic medication adherence (Day et al. 1995; Gray et al. 2008; Bock et al. 2020; Waddell and Taylor 2008). Although these instruments have substantial value of important clinical parameters and can help in determining treatment approaches, the current side‐effect questionnaires focus on younger to middle‐aged adult populations. Consequently, existing instruments miss older age‐specific side effects, including cognitive side effects, functional decline (e.g., impaired hearing and vision), and somatic complications. Furthermore, these instruments were yet to be validated in Dutch.

In this study, we originally developed the Scale for Somatic and Cognitive Complaints of Psychotropic Medication Adult‐Aged‐Spectrum (SCOPA) to fill the gap by investigating these older age‐specific side effects in older adult mental health patients (Supporting Information S1: Appendix 2; Serretti and Mandelli 2010). Hence, the field will benefit from an improved measure that includes topics applicable to the full adult‐aged spectrum to improve monitoring and treatment decisions across the full adult‐aged spectrum. The overall aim of the current study was to *develop* and *validate* the psychometric properties (internal consistency and construct validity) of the SCOPA in a Dutch general population subsample and in a mental health patient subsample.

## 2 Materials and Methods

### 2.1 Item and Scale Development

The SCOPA was constructed based on the most common patient‐reported side effects from psychotropic medication and on clinical expertise in pharmacological treatment following a literature study. Further scale development took place that involved critical assessment by a panel of experienced clinicians (including a medical doctor, geriatric psychiatrist, clinical geriatrician, and clinical pharmacologist). Items concerning cognitive complaints, falling incidents, and sexual dysfunction were added in collaboration with the expert panel, making the scales applicable for the full age spectrum.

The Dutch questionnaire was translated to English through a multidisciplinary review of researchers. An official translator was available for consultation when there was reasonable doubt concerning the formal English translation (Supporting Information S2: Appendix 2).

The section on side effects contained 55 items grouped into categories of side effects pertaining to different ‘organ’ systems: respectively, the nervous system, suicidality, cardiovascular system, musculoskeletal system, gastrointestinal system, urinary and reproductive system, perception, and integumentary system. Participants indicated per item the degree to which they experienced a side effect in the past 2 weeks. Responses were provided on a five‐point Likert scale (informal English translation is “not at all”, “a bit”, “somewhat”, “a lot”, “extremely”). A score of zero (“not at all”) referred to the absolute absence of the complaint in the past two weeks. A higher score indicated more severe interference of a particular side effect with daily life functioning. In addition, complications because of experiencing side effects were recorded (i.e., changes in body weight, syncope, falls, traffic accidents, hospitalisations, general practitioner consultation, and disabilities in activities of daily living). Also, names of psychotropic medication, doses, dosing frequencies, indications, start dates, adherence to medication, age, sex, and measurements of height and weight were collected.

The questionnaire could be filled in without professional assistance and was typically completed in 10 min. Part of the sample completed the SCOPA after the answer options underwent a subtle change in wording. The answer option “average” was replaced by “somewhat” (in Dutch: “een beetje”) due to ambiguous interpretations in the preliminary stages of data collection. The SCOPA was available as a paper questionnaire as well as an online survey.

### 2.2 Participants

The total sample consisted of a subsample of in‐ and outpatients from healthcare institutes and a general population subsample. The total sample consisted of 487 Dutch‐ and English‐speaking participants, ranging 18–87 years old (*M*_*age*_ = 48.07, SD = 21.9). Of the total sample size, 36.8% (*n* = 179) were male, 58.9% (*n* = 287) were female, and 4.3% (*n* = 21) had an unknown sex. Participants were labelled as “unknown sex” if information about their sex was missing. The convenience sample of in‐ and outpatients aged 18 years and older was recruited through a multi‐centre design from various divisions of the Mondriaan Mental Health Centre Transition Care Clinic (*n* = 7), Department of Old Age Psychiatry, including the Clinical Centre of Excellence for Older Adults with Personality Disorders (*n* = 112), Flexible Assertive Community Treatment (FACT) (*n* = 1), long‐stay wards for younger adults (*n* = 20), Breburg Department of Old Age Psychiatry (*n* = 9), and the De Viersprong Mental Healthcare Institute in the Netherlands (*n* = 57).

Participants in the in‐ and outpatient sample were recruited by their therapist or the researcher directly. Methods used in the recruitment process were word‐of‐mouth advertising, the use of social media, and leafleting at the abovementioned institutions. Eligibility for participation required patients to be older than 18 years, to be in treatment in mental healthcare, and to be willing and able to give informed consent. Subjects were excluded from the study if they were diagnosed with major cognitive disorders like moderate to severe dementia, were not able to participate due to physical handicaps, or were insufficiently resilient or stable as determined by the patient's therapist. Participants in the in‐ and outpatient subsample had been diagnosed with a mental health disorder by a licensed psychiatrist or psychologist who used the criteria of the Diagnostic and Statistical Manual of Mental Disorders (First 2013). Possible diagnoses were personality, neurodevelopmental, psychotic, bipolar, anxiety, obsessive‐compulsive, somatoform, substance abuse, eating, cognitive, impulse control, or sleeping disorders, and trauma, depressive syndromes, or gender dysphoria. Patient‐specific psychiatric diagnoses were not of importance in this study. Psychiatric diagnoses were checked against the electronic patient file and diagnoses were reviewed in multidisciplinary teams.

In the general population subsample, the same methods as in the in‐ and outpatient sample were used during the recruitment process. Moreover, additional general population controls were recruited through an online survey system, through which students from the Faculty of Psychology and Neuroscience from Maastricht University could participate.

### 2.3 Procedures

The data were collected between March 2019 and May 2023, using a cross‐sectional survey study design. No follow‐up occurred. The study was approved by the Medical Ethical Committee Zuyderland (METC‐Z) and was not bound to the “Wet Medisch‐Wetenschappelijk Onderzoek met Mensen” (WMO; Medical Research Involving Human Subjects Act). Informed consent was obtained prior to participation. Patients expected to be incapable of completing the questionnaire autonomously were helped to complete the questionnaire by providing verbal explanations for the definition of complaints.

### 2.4 Instruments

To assess subjective side effects of psychotropic medication, the SCOPA was used. Background variables such as date of assessment, diagnoses of mental health disorders, somatic disorders, and demographic characteristics of participants, including age and sex, were recorded. If data were missing from the questionnaire, they were extracted from the patients' electronic medical records after collecting the patients' permission. Details of prescriptions of psychotropic and somatic medication were collected and verified with the patients' electronic medication management system.

### 2.5 Statistical Analysis

Descriptive statistics were used to analyse sample characteristics. Analyses were performed with SPSS v.28.0 (IBM‐SPSS Inc., Chicago, IL, USA). Little's (1988) MCAR test was used to assess whether missing data were missing completely at random. The internal consistency of the different subscales in the total sample was estimated with average inter‐item correlations (AICs). An AIC is independent of the number of items in the scale and can handle missing data. An AIC between 0.15 and 0.50 is considered as adequate internal consistency (Clark and Watson 1995). Cronbach's alphas (Cαs) are also reported for the different subscales. Alphas between 0.7 and 0.9 are considered good. The minimal acceptable alpha is considered 0.6 (Tavakol and Dennick 2011). In this study, we originally developed the SCOPA based on a theoretical construct we had empirically tested.

Given the explorative nature of this study, explanatory factor analysis (EFA) was chosen over confirmatory factor analysis (CFA). CFA was not retested with the same sample to prevent bias due to overfitting. The number of factors was determined with parallel analysis and scree plots. Factor correlations were assessed to determine the appropriate type of rotation. Oblique (*promax*) rotation was applied because the latent factors were related. The rotated factor structure was considered to identify weak factor‐loadings (lower than 0.3). Scales were considered to load significantly on a factor if the factor‐loading was at least 0.45 (Comrey and Lee 2013).

### 2.6 Sample Size

Before conducting the study, the aimed at patient sample size as well as the control sample size was determined to be at least 100 participants (Kyriazos 2018). Sample size adequacy was planned to be determined by evaluation of communalities of the items (i.e., the amount of variance in an item accounted for by a factor) and the strengths of factor‐loadings (Kyriazos 2018; MacCallum et al. 1999). Strong data are characterized by consistently high communalities, high factor‐loadings on at least three items (i.e., factor overdetermination), and a low number of extracted factors (MacCallum et al. 1999; Osborne and Costello 2004; Preacher and MacCallum 2002).

## 3 Results

### 3.1 Demographics and Clinical Characteristics

Table 1 shows an overview of the demographic and clinical characteristics of the sample. Primary Diagnostic and Statistical Manual of Mental Disorders (DSM‐5) diagnoses of the in‐ and outpatient sample were recorded; 72.3% (*n* = 149) of the sample were diagnosed with a personality disorder, 13.6% (*n* = 28) with a psychotic disorder, 46.6% (*n* = 96) with a mood disorder, 7.3% (*n* = 15) with a neurodevelopmental disorder, 20.9% (*n* = 43) with an anxiety disorder, 18.9% (*n* = 39) with a traumatic disorder, 16.5% (*n* = 34) with an addiction, and 8.3% (*n* = 17) with a cognitive impairment. Eighty‐two percent (*n* = 169) of the patient sample had more than one DSM‐5 diagnosis. Somatic disorders of the patient sample were recorded: 46.1% (*n* = 95) of the subsample had internal diseases, 32.5% (*n* = 67) cardiovascular diseases, 19.9% (*n* = 41) neurological diseases, 19.9% (*n* = 41) orthopaedic diseases, 14.1% (*n* = 29) pulmonary diseases, and 7.3% (*n* = 15) ear, nose, and throat conditions. The age distributions of the in‐ and outpatient and general population samples were widespread, covering the full age spectrum (ages 19–87 years).

**TABLE 1:** Demographic and clinical characteristics of the total sample.

	In‐ and outpatient sample			General population sample		
	Total sample (*n* = 206)	Adults^a^ (*n* = 84)	Older adults^b^ (*n* = 122)	Total sample (*n* = 281)	Adults (*n* = 177)	Older adults (*n* = 104)
Age in years, mean (SD), range	56.74 (18.05), 19‐85	37.88 (12.07), 19‐59	69.73 (5.73), 61‐85	41.72 (22.3), 18‐87	26.43 (11.44), 18‐59	67.74 (6.83), 60‐87
Sex, *n* (%)						
Male	89 (43.2%)	37 (44.0%)	52 (42.6%)	90 (32.0%)	40 (22.6%)	50 (48.1%)
Female	117 (56.8%)	47 (56.0%)	70 (57.4%)	170 (60.5%)	118 (66.7%)	52 (50.0%)
Unknown	0 (0.0%)	0 (0.0%)	0 (0.0%)	21 (7.5%)	19 (10.7%)	2 (1.9%)
Sample location						
Inpatient sample	204 (99%)	84 (100%)	120 (98.4%)	110 (39.1%)	34 (19.2%)	76 (73.1%)
Outpatient sample	2 (1.0%)	0 (0.0%)	2 (1.6%)	171 (60.9%)	143 (80.8%)	28 (26.9%)
Prescription of psychotropic medication, *n* (%)	158 (76.7%)	58 (69.0%)	100 (82.0%)	9 (3.2%)	2 (1.1%)	7 (6.7%)
Number of prescribed psychotropic medications, mean (SD), range	1.62 (1.32) 0‐6	1.38 (1.29)	1.81 (1.34) 0‐6	0.04 (0.229)	0.01 (0.106) 0‐1	0.09 (0.346) 0‐2

*Note:* This table provides an overview of demographic and clinical characteristics of the in‐ and outpatient sample, the general population sample, and for adults and older adults separately. Sample sizes (n) are shown with percentages (%) or standard deviations (*SD*s).

^a^ Adult = age from 18 until 64 years.

^b^ Older adult = age from 65 until 99 years.

### 3.2 Missing Value Analysis

Participants who had > 20% missing data were removed from analyses, as such amounts of missing data signify unreliable response tendencies and inclusion would bias estimates of the factor‐loadings (*n* = 1). In total, 0.36% of data were missing. Twenty‐two individuals had one (2.3% of the total sample) or two (2.3% of the total sample) items missing (*n* = 11 with one item missing; *n* = 11 with two items missing). Further rates of missing data were low. Little's MCAR tests indicated that data were not missing completely at random (*χ* ^*2*^ = 1364.31; *p* < 0.0001). This was driven by three items: orgasmic dysfunction (*n* = 15), dyspareunia (*n* = 16), and erectile dysfunction (*n* = 8). Therefore, missing data were replaced by item‐mean imputation since substitution with the participants' mean would bias the results (Kang 2013). Rates of missing data were low, item‐mean imputation did not influence the expected outcomes, and factor analysis was performed with pairwise case exclusion (i.e., cases with missing values were included in the analysis).

### 3.3 Internal Consistency

The internal consistency of the SCOPA was calculated for the different theoretically based subscales through Cronbach's alphas and AIC values. The item ‘erectile dysfunction’ was not included due to the gender‐specific response format which biased the results. Table 2 shows an overview of the SCOPA's internal consistency. All correlations are significant at the level *p* < 0.001. All AIC values were above the minimum required level of 0.15, except the gastro‐intestinal and urogenital/reproductive subscales of the in‐ and outpatient subsample, with AICs of 0.141 and 0.137, respectively. The Cronbach's alphas varied between 0.256 and 0.856.

**TABLE 2:** Scales, number of items per scale, and internal consistency of the total sample.

		Total sample		In‐ and outpatient subsample		General population subsample	
Scales	Number of items	Ca	AIC	Ca	AIC	Ca	AIC
Nervous system	12	0.856	0.303	0.815	0.243	0.827	0.268
Suicidality	3	0.488	0.257	0.256	0.255	0.385	0.393
Cardiovascular system	6	0.755	0.309	0.730	0.313	0.640	0.232
Musculoskeletal system	8	0.827	0.400	0.825	0.372	0.739	0.251
Gastrointestinal system	8	0.694	0.219	0.569	0.141	0.698	0.227
Urinary/reproductive system	8	0.615	0.175	0.556	0.137	0.589	0.170
Perception	7	0.694	0.253	0.644	0.204	0.654	0.243
Integumentary system	4	0.627	0.301	0.593	0.274	0.636	0.309

*Note:* This table provides an overview of different subscales, number of items per scale, and the internal consistencies measured with Cronbach's alpha (Ca) and the average inter‐item correlations (AICs).

### 3.4 Construct Validity

Multivariate assumptions of EFA were met. Factor structure was analysed separately between the patient sample and the general population sample. The item ‘erectile dysfunction’ was not included in the EFA since this complaint is applicable to male patients only, and for separate reliable factor analysis a larger sample of male participants would be required. Items showing zero variance were excluded from the analysis. Also, dichotomic items (the items ‘epileptic insult’ and ‘suicide attempt’) were excluded from the analysis since the dichotomic response format would bias the results. Table 3 shows the factor‐loadings of the different items in the general population and patient subsamples.

**TABLE 3:** Rotated component matrix for EFA.

	In‐ and outpatient sample		General population sample	
	Component		Component	
	1	2	1	2
1. Difficulty falling asleep	0.040	**0.557**	**0.594**	−0.031
2. Difficulty sleeping through	0.256	**0.317**	**0.410**	−0.020
3. Forgetfulness: Short‐term memory problems	0.084	**0.565**	**0.491**	0.137
4. Forgetfulness: Long‐term memory problems	−0.020	**0.556**	**0.515**	0.111
5. Confusion: Confusion about time, places, persons, or events	0.243	**0.425**	0.104	**0.543**
6. Difficulty concentrating	0.006	**0.703**	**0.666**	0.016
7. Headaches	**0.356**	0.163	**0.472**	0.028
8. Tense or restless feeling in the body	0.053	**0.683**	**0.758**	−0.053
9. Irritable and/or aggressive	−0.067	**0.660**	**0.687**	−0.122
10. Overly excited and/or cheerful mood	0.011	0.135	0.246	0.076
11. Depressed mood	−0.173	**0.800**	**0.822**	−0.091
12. (Increased) feelings of wanting to harm yourself	−0.145	**0.627**	**0.650**	−0.322
13. (Increased) thoughts about death	−0.182	**0.761**	**0.628**	0.023
14. Tight feeling of the chest	**0.447**	0.187	**0.452**	0.074
15. Heart palpitations	**0.310**	**0.302**	**0.598**	−0.179
16. Shortness of breath on slight exertion	**0.522**	0.095	**0.433**	0.164
17. Swollen feet or ankles	**0.570**	−0.066	−0.166	**0.666**
18. Badly healing wounds	**0.521**	−0.019	0.285	0.098
19. Easier or longer bleeding from wounds	**0.529**	−0.096	0.158	**0.315**
20. Dizziness	**0.607**	−0.024	**0.383**	0.233
21. Tingling sensation	**0.630**	0.024	**0.409**	0.226
22. Decreased muscle strength	**0.684**	−0.040	0.244	**0.397**
23. Feeling of stiffness in muscles	**0.622**	0.091	0.197	**0.451**
24. Muscle ache	**0.629**	0.043	0.245	**0.342**
25. Sensation of uncontrollable movement of muscles	**0.592**	0.024	**0.328**	−0.153
26. Restless legs at night	**0.390**	0.233	0.084	**0.384**
27. Urge to walk	0.266	0.259	**0.467**	0.022
28. Increased appetite	**0.324**	−0.050	**0.378**	−0.026
29. Decreased appetite	−0.730	**0.488**	**0.376**	0.225
30. Nausea	**0.353**	0.098	**0.468**	0.010
31. Difficulty defecating	0.268	0.175	0.020	**0.536**
32. Diarrhoea	0.180	0.199	**0.395**	0.036
33. Excessive thirst, drinking a lot	**0.410**	0.024	**0.355**	0.152
34. Dry mouth	**0.364**	0.169	**0.589**	0.037
35. Salivation	**0.404**	−0.161	−0.084	**0.508**
36. Breast Formation	**0.467**	−0.195	0.103	**0.313**
37. Frequent urination	**0.376**	0.019	0.085	**0.410**
38. Pain during urination	−0.052	**0.327**	−0.098	**0.596**
39. Difficulty urinating	0.249	0.113	−0.002	0.251
40. Increased sexual desire	0.118	0.029	0.114	**0.323**
41. Decreased sexual desire	−0.019	**0.555**	0.251	0.002
42. Pain during sexual contact	0.192	0.241	−0.026	**0.359**
43. Orgasmic dysfunction	−0.188	**0.575**	0.118	0.256
44. Hearing impairment	**0.417**	−0.078	−0.138	**0.562**
45. Beep or ringing sound in one or both ears	**0.404**	−0.057	−0.182	**0.502**
46. Blurred vision	**0.542**	−0.009	0.143	**0.504**
47. Double vision	**0.428**	−0.011	−0.043	**0.560**
48. Loss of voice	**0.418**	−0.089	0.088	**0.312**
49. Changed taste	**0.412**	0.000	−0.231	**0.724**
50. Hallucinations	0.185	0.180	−0.027	**0.582**
51. Itching	**0.420**	0.028	0.232	0.292
52. Skin rash	**0.533**	−0.016	0.215	0.155
53. Sweating	**0.253**	**0.369**	0.205	**0.309**
54. Hair loss	**0.310**	0.047	0.231	0.205

*Note:* Extraction method: principal component analysis. Rotation method: promax with Kaiser normalization, three iterations.

First, we analysed the construct validity of the general population subsample. Scree‐plot inspection likewise suggested that two or three factors would suffice. A two‐factor structure was hypothesized. Oblique rotation showed mutual correlation between factors 1 and 2 (*r* = 0.546). Hence, oblique rotation (*promax*) was recommended for EFA. The two extracted factors cumulatively explained a total of 25.463% of the item variance. The Kaiser‐Meyer‐Olkin measure indicated moderate factorability of the correlation matrix (overall MSA = 0.822) (Kaiser 1974). Bartlett's test of sphericity was also significant, *χ* ^*2*^(1431) = 6056.385; *p* < 0.001, suggesting correlations between items were statistically significantly different from zero (Bartlett 1954). Eight items had low factor‐loadings (< 0.3), suggesting that these items extracted little variance for any of the factors. Items with low factor‐loadings or cross‐loadings were removed before EFA was conducted (*n* = 8, *n* = 0, respectively).

Second, we analysed the construct validity of the in‐ and outpatient subsamples. Scree‐plot inspection likewise suggested that two or three factors would be adequate. A two‐factor structure was hypothesized. Oblique rotation showed mutual correlation between factors 1 and 2 (*r* = 0.546). Hence, oblique rotation (*promax*) was recommended for EFA. The two extracted factors cumulatively explained a total of 25.412% of the item variance. The Kaiser‐Meyer‐Olkin measure indicated moderate factorability of the correlation matrix (overall MSA = 0.797) (Kaiser 1974). Bartlett's test of sphericity was also significant, *χ* ^*2*^(1431) = 4317.432; *p* < 0.001, suggesting correlations between items were statistically significantly different from zero (Bartlett 1954). Eight items had low factor‐loadings (< 0.3), suggesting that these items extracted little variance for any of the factors. One item cross‐loaded significantly on both constructs. Items with low factor‐loadings or cross‐loadings were removed before EFA (*n* = 7, *n* = 1, respectively). The two factors found in both subsamples were hypothesised to represent two aspects of side effects, that is, of *somatic* and *psychic* nature. Following oblique rotation, the two factors explained 20.263% and 25.463% or 20.579% and 25.412% of the item variance, respectively.

### 3.5 Subsample Differences

Subsample differences on the scales of the SCOPA were assessed by *t*‐tests for independent samples for both the in‐ and outpatient subsample and the general population subsample. In all subscales, the in‐ and outpatient subgroup responded at least 25% higher than the general population subgroup. Table 4 gives an overview of the SCOPA subsample differences.

**TABLE 4:** Means and standard deviations on the SCOPA's subscales.

Scales	Total mean (SD)	In‐ and outpatient means (SD)	General population mean (SD)
Nervous system	10.19 (7.507)	14.47 (7.468)	7.07 (5.819)
Suicidality	0.83 (1.448)	1.46 (1.786)	0.37 (0.898)
Cardiovascular system	2.68 (3.325)	4.33 (3.392)	1.48 (2.037)
Musculoskeletal system	5.06 (5.223)	7.90 (6.020)	2.99 (3.262)
Gastrointestinal system	4.08 (4.025)	6.20 (4.278)	2.54 (3.009)
Urinary and reproductive system	3.20 (3.439)	4.66 (3.917)	2.13 (2.563)
Perception	2.39 (3.056)	3.74 (3.563)	1.41 (2.150)
Integumentary system	2.39 (2.528)	2.98 (2.771)	1.96 (2.244)

*Note:* This table provides an overview of the means and standard deviations (SDs) of the in‐ and outpatient sample and general population sample on the SCOPA's scales.

### 3.6 Complication Prevalence

The following complications of the total sample, in‐ and outpatient subsample, and general population subsample were collected and appear in Table 5. In addition, in the in‐ and outpatient subsample, 27.5% (*n* = 33) of the older adults (age > 60 years) reported weight gain or loss, 9.2% (*n* = 11) reported falling, 1.7% (*n* = 2) reported syncope, 0.8% (*n* = 1) reported a traffic accident, 9.2% (*n* = 11) reported a general practitioner consultation, and 0.8% (*n* = 1) reported a hospital admission. Whereas 28.2% (*n* = 24) of the adult subsample (age < 60 years) reported weight gain or loss, 2.4% (*n* = 2) reported falling, 2.4% (*n* = 2) reported syncope, 2.4% (*n* = 2) reported a general practitioner consultation, 2.4% (*n* = 2) reported a hospital admission, and no traffic accidents were reported.

**TABLE 5:** Frequencies of reported complications in the total sample.

Complications	Total sample, *n* (%)	In‐ and outpatient subsample, *n* (%)	General population subsample, *n* (%)
Weight gain or loss	75 (15.4%)	57 (27.8%)	18 (6.4%)
Fall	16 (3.3%)	13 (6.3%)	3 (1.1%)
Syncope	7 (1.4%)	4 (2.0%)	3 (1.1%)
Traffic accident	4 (0.8%)	1 (0.5%)	3 (1.1%)
General practitioner consultation	19 (3.9%)	13 (6.3%)	6 (2.1%)
Hospital admission	4 (0.8%)	3 (1.5%)	1 (0.4%)
Epileptic insult	5 (1.0%)	2 (1.0%)	3 (1.1%)
Suicide attempt	2 (0.4%)	2 (1.0%)	0 (0.0%)
Other	40 (8.2%)	33 (16.1%)	7 (2.5%)

*Note:* Prevalence of complications across the total sample and across the in‐ and outpatient and general population subsamples reported separately, expressed in frequencies (n) and percentages (%).

## 4 Discussion

This study was a *first insight* into evaluating the newly developed SCOPA for its psychometric properties in the assessment of side effects in a Dutch general population subsample and mental healthcare in‐ and outpatient sample. Reliability analysis was overall robust and confirmed moderate to good internal consistency of the theoretically based subscales of the SCOPA, and EFA supported a possible two‐factor solution (representing two overshadowing constructs of complaints, i.e., of *somatic* and *psychic* nature). The SCOPA's practical implications as a standardized approach to screen for side effects in clinical practice are feasible due to several reasons. First, completion of the questionnaire is convenient: It requires about 10 min and no supervision of a healthcare professional. Second, the combined response format leaves room for both the severity of complaints in specific organ domains as well as experienced complications due to medication use. Third, a completed questionnaire can be evaluated with the patient during treatment, giving space to speak more freely about complaints that have been flying under the radar previously, thus contributing to potential over‐time side‐effect monitoring and a patient‐centred treatment regimen. Finally, the SCOPA is available in both Dutch and English, making it a useful tool for a broad spectrum of patients in different clinical settings. The SCOPA seeks to address the need for a standardized approach to the assessment of the experience of side effects across a wide age range. In doing so, the current validation study had several strengths. First, the SCOPA was validated with a representative, diverse sample of patients in mental healthcare using medication, concurrent with the way the instrument is intended to be used after validation. Second, the SCOPA was concomitantly validated with a general population control sample with general medication use, which underlined the outcomes of the reliability and factor analysis from the in‐ and outpatient subsample. Third, by including older adult‐specific items, it is the *first instrument* in its class designed for application in both adult and older adult populations. Last, the SCOPA promotes reports of complaints by inquiring about patients' experience of complaints, without solely relying on spontaneous report.

Still, the results of this study should be seen in the light of four limitations. First, this was a first psychometric study: How the SCOPA compares to other objective questionnaires of side effects (*criterion validity*) was not assessed and poses a necessary target for future research. In addition, convergent and discriminant validity could be assessed by analysing the relationship of the SCOPA's dimensions with various measures of health and objective measures of side effects. Second, it was hypothesized that disorder‐related symptoms at the time of assessment may influence the report of side effects (Spink et al. 2018). However, symptoms at time of assessment were not reported. Comorbid issues independent of psychotropic medication use (e.g., clinical manifestations and symptoms) pose an important challenge to future estimations of cause‐effect relationships between medication and side effects and complications. Moreover, previous research has shown that sex, age, and the duration of psychotropic medication use can affect side‐effect expression and report (Ferguson 2001; Gandhi et al. 2004; Steckenrider 2023). These were not specific aims of this study. Third, our general population subsample was not screened for psychiatric comorbidity and the use of psychotropic medication was not an exclusion criterion. Therefore, a part of the general population subsample included controls with a psychiatric DSM‐5 diagnosis, which could have influenced response tendencies. Fourth, different baseline characteristics (e.g., the number and dosage of psychotropic medication used) between the two subsamples may have influenced the overall factor structure (Ferguson 2001; Steckenrider 2023).

Our internal reliability analysis provided Cronbach's alphas varying between 0.256 and 0.856. In the in‐ and outpatient subsample, four of eight subscales had Cronbach's alphas lower than the acceptable value of 0.6 (Tavakol and Dennick 2011), possibly explained by the low number of items on these specific subscale (suicidality, *n* = 3; integumentary, *n* = 4) or the duality or ambiguity in the subscales (gastrological and intestinal items, urological and genital items) (Taber 2017). Determination of the accuracy of the extracted factor structure suggested by EFA involves the evaluation of sample size. Rules of thumb for determining and evaluating sample size in factor analysis are considered imprecise and possibly inappropriate. Instead, MacCallum et al. (1999) concluded that sufficiently strong data justify the use of factor analysis and allows the drawing of conclusions based on it even with small samples or small observation‐to‐item ratios. Low communalities (and overall variances), like in the current analysis (< 0.5), can hinder accurate estimation of factor‐loadings (Hogarty et al. 2005; Mundfrom et al. 2005), like in the general population subsample, 12 of the somatic questionnaire items loaded onto the psychic factor. Nevertheless, the EFA in the current study suggested hopefulness in that it attained good extraction of factors, since the analysis proposed well‐determined factors with an independently strong explanation of the factor structure. Differences in factor‐loading could be explained through the relation between somatic and psychiatric complaints (Mostafaei et al. 2019; Seiffge‐Krenke and Sattel 2024).

Overall, further cross‐validation of the SCOPA is necessary. It should involve retesting the internal consistencies, replicating the two‐factor structure with CFA, seeking divergent and convergent validity, and providing additional cross‐validation studies. Moreover, an English version of the SCOPA might be validated to externalize the SCOPA as an international tool for monitoring side effects.

## 5 Conclusions

With this first‐study we inquired into the psychometric quality of the SCOPA, showing that the SCOPA can differentiate between psychic and somatic constructs; these factors showed a moderate to good internal consistency per the theoretically based subscales. Hence, with the development of instruments to assess complaints caused by psychotropic medication, the field is in pursuit of improved patient safety. Ultimately, the SCOPA aims to contribute to reliable, practical, and valid monitoring of complaints and complications of psychotropic medication. Its potential as a tool for monitoring side effects over time requires further investigation, however.

## Conflicts of Interest

The authors declare no conflicts of interest.

## Supporting information

Supporting Information S1

Supporting Information S2

Supporting Information S3

## Data Availability

The datasets generated during and/or analysed during the current study are not publicly available but are available from the corresponding author on reasonable request. The author(s) received no financial support for the research, authorship, and/or publication of this article.
