# Supplementary material for: Psychometric Properties of the Scale for Subjective Somatic and Cognitive Complaints of Psychotropic Medication Adult‐Aged‐Spectrum (SCOPA)
Source: Hum Psychopharmacol. 2025 Jul 9;40(4):e70009. doi: 10.1002/hup.70009 (PMC12240232; doi:10.1002/hup.70009)
Supplement: Supplementary file 1 — Supporting Information S1 [file HUP-40-e70009-s002.pdf]

Bijwerkingen vragenlijst
Somatic and Cognitive Complaints of Psychotropic Medication Adult-Aged-Spectrum (SCOPA)

Datum: …………………….

Naam: ………………………………………… Man / Vrouw*

Geboortedatum: ………………………………

Deel A

Lengte (cm)	
Gewicht (kg)	

Actuele medicatie

	Naam medicijn	Dosering (mg)	Aantal tabletten per dag	Voor welke reden neemt u dit medicijn?	Startdatum (ongeveer)
1					
2					
3					
4					
5					
6					
7					
8					
9					
10					
11					

12					
13					
14					
15					

Deel B

Therapietrouw

Hoe beoordeelt u uw therapietrouw?*

- Goed: u neemt trouw uw medicatie in op de voorgeschreven manier en momenten en vergeet het zelden of nooit.
- Matig: u neemt *meestal wel* uw medicatie in op de voorgeschreven manier en momenten, maar vergeet het ook regelmatig.
- Slecht: u neemt *meestal niet* uw medicatie in op de voorgeschreven manier en momenten.
- U neemt helemaal geen medicatie in die door de arts is voorgeschreven.

* *Vink aan wat van toepassing is*

Huidige klachten

Het gaat om de bijwerkingen/klachten die aanwezig waren, of zijn veranderd in de *afgelopen 2 weken*.

Omcirkel het meest passende cijfer. (0 = helemaal geen last van, 4 = heel erg veel last van)

Zenuwstelsel

1. Problemen met inslapen

Helemaal niet	Een beetje	Nogal	Veel	Extreem
0	1	2	3	4

2. Problemen met doorslapen

Helemaal niet	Een beetje	Nogal	Veel	Extreem
0	1	2	3	4

3. Vergeetachtigheid: problemen met het kortetermijngeheugen

Helemaal niet	Een beetje	Nogal	Veel	Extreem
0	1	2	3	4

4. Vergeetachtigheid: problemen met het langetermijngeheugen

Helemaal niet	Een beetje	Nogal	Veel	Extreem
0	1	2	3	4

5. Verwardheid: verwarringen in tijd, plaats, persoon of gebeurtenis

Helemaal niet	Een beetje	Nogal	Veel	Extreem
0	1	2	3	4

6. Minder goed kunnen concentreren (bijv. tijdens lezen, televisie kijken of een gesprek)

Helemaal niet	Een beetje	Nogal	Veel	Extreem
0	1	2	3	4

7. Hoofdpijn

Helemaal niet	Een beetje	Nogal	Veel	Extreem
0	1	2	3	4

8. Gespannen of onrustig gevoel in het lichaam

Helemaal niet	Een beetje	Nogal	Veel	Extreem
0	1	2	3	4

9. Snel geïrriteerd en/of agressief

Helemaal niet	Een beetje	Nogal	Veel	Extreem
0	1	2	3	4

10. Overmatig opgewekte en/of vrolijke stemming

Helemaal niet	Een beetje	Nogal	Veel	Extreem
0	1	2	3	4

11. Sombere stemming

Helemaal niet	Een beetje	Nogal	Veel	Extreem
0	1	2	3	4

12. Epileptische aanval

Ja Nee

Suïcidaliteit (zelfmoordneigingen)

13. (Toename) van gevoelens om uzelf te willen verwonden

Helemaal niet	Een beetje	Nogal	Veel	Extreem
0	1	2	3	4

14. (Toename) van gedachten aan de dood

Helemaal niet	Een beetje	Nogal	Veel	Extreem
0	1	2	3	4

15. Heeft u in de afgelopen 2 weken een poging gedaan om een einde aan uw leven te maken?

Ja Nee

Hart- en vaatstelsel

16. Zwaar of drukkend gevoel op de borst

Helemaal niet	Een beetje	Nogal	Veel	Extreem
0	1	2	3	4

17. Hartkloppingen

Helemaal niet	Een beetje	Nogal	Veel	Extreem
0	1	2	3	4

18. Kortademigheid bij geringe inspanning

Helemaal niet	Een beetje	Nogal	Veel	Extreem
0	1	2	3	4

19. Gezwollen voeten of enkels

Helemaal niet	Een beetje	Nogal	Veel	Extreem
0	1	2	3	4

20. Slecht genezende wondjes

Helemaal niet	Een beetje	Nogal	Veel	Extreem
0	1	2	3	4

21. Makkelijker of langer bloeden uit wondjes

Helemaal niet	Een beetje	Nogal	Veel	Extreem
0	1	2	3	4

Bewegingsapparaat

22. Duizeligheid

Helemaal niet	Een beetje	Nogal	Veel	Extreem
0	1	2	3	4

23. Tintelend gevoel (o.a. armen, benen, handen, voeten)

Helemaal niet	Een beetje	Nogal	Veel	Extreem
0	1	2	3	4

24. Verminderde kracht in spieren (o.a. armen, benen)

Helemaal niet	Een beetje	Nogal	Veel	Extreem
0	1	2	3	4

25. Gevoel van stijfheid in spieren (o.a. armen, benen)

Helemaal niet	Een beetje	Nogal	Veel	Extreem
0	1	2	3	4

26. Spierpijn

Helemaal niet	Een beetje	Nogal	Veel	Extreem
0	1	2	3	4

27. Gevoel van onbeheersbare beweging van spieren (o.a. ogen, lippen, nek, armen, benen, handen, voeten)

Helemaal niet	Een beetje	Nogal	Veel	Extreem
0	1	2	3	4

28. Onrustig gevoel in de benen ’s nachts

Helemaal niet	Een beetje	Nogal	Veel	Extreem
0	1	2	3	4

29. Loopdrang: het gevoel dat u moet blijven lopen of moeilijk stil kan blijven zitten

Helemaal niet	Een beetje	Nogal	Veel	Extreem
0	1	2	3	4

Maag- en darmstelsel

30. Toename van de eetlust

Helemaal niet	Een beetje	Nogal	Veel	Extreem
0	1	2	3	4

31. Afname van de eetlust

Helemaal niet	Een beetje	Nogal	Veel	Extreem
0	1	2	3	4

32. Misselijkheid

Helemaal niet	Een beetje	Nogal	Veel	Extreem
0	1	2	3	4

33. Moeite met ontlasting maken (opstopping in de darmen)

Helemaal niet	Een beetje	Nogal	Veel	Extreem
0	1	2	3	4

34. Diarree

Helemaal niet	Een beetje	Nogal	Veel	Extreem
0	1	2	3	4

35. Overmatige dorst, veel drinken

Helemaal niet	Een beetje	Nogal	Veel	Extreem
0	1	2	3	4

36. Droge mond

Helemaal niet	Een beetje	Nogal	Veel	Extreem
0	1	2	3	4

37. Speekselvloed

Helemaal niet	Een beetje	Nogal	Veel	Extreem
0	1	2	3	4

<u>Urine- en voortplantingsstelsel</u>

38. (Toename van) borstvorming

Helemaal niet	Een beetje	Nogal	Veel	Extreem
0	1	2	3	4

39. Veel plassen

Helemaal niet	Een beetje	Nogal	Veel	Extreem
0	1	2	3	4

40. Pijn bij het plassen

Helemaal niet	Een beetje	Nogal	Veel	Extreem
0	1	2	3	4

41. Moeite met plassen (o.a. slechte straal, druppeltjes)

Helemaal niet	Een beetje	Nogal	Veel	Extreem
0	1	2	3	4

42. Toename seksueel verlangen

Helemaal niet	Een beetje	Nogal	Veel	Extreem
0	1	2	3	4

43. Afname seksueel verlangen

Helemaal niet	Een beetje	Nogal	Veel	Extreem
0	1	2	3	4

44. Pijn bij seksueel contact

Helemaal niet	Een beetje	Nogal	Veel	Extreem
0	1	2	3	4

45. Niet kunnen klaarkomen

Helemaal niet	Een beetje	Nogal	Veel	Extreem
0	1	2	3	4

46. Voor mannen: geen erectie kunnen krijgen. *Als u een vrouw bent, sla deze vraag over.*

Helemaal niet	Een beetje	Nogal	Veel	Extreem
0	1	2	3	4

Waarnemen

47. Verslechtering van het gehoor

Helemaal niet	Een beetje	Nogal	Veel	Extreem
0	1	2	3	4

48. Een pieptoon of suizen in één of beide oren

Helemaal niet	Een beetje	Nogal	Veel	Extreem
0	1	2	3	4

49. Wazig zien

Helemaal niet	Een beetje	Nogal	Veel	Extreem
0	1	2	3	4

50. Dubbel zien

Helemaal niet	Een beetje	Nogal	Veel	Extreem
0	1	2	3	4

51. Verlies van de stem

Helemaal niet	Een beetje	Nogal	Veel	Extreem
0	1	2	3	4

52. Veranderde smaak

Helemaal niet	Een beetje	Nogal	Veel	Extreem
0	1	2	3	4

53. Het horen, zien, ruiken, voelen of proeven van dingen die andere mensen niet ervaren

Helemaal niet	Een beetje	Nogal	Veel	Extreem
0	1	2	3	4

Huid

54. Jeuk

Helemaal niet	Een beetje	Nogal	Veel	Extreem
0	1	2	3	4

55. Huiduitslag

Helemaal niet	Een beetje	Nogal	Veel	Extreem
0	1	2	3	4

56. Zweten

Helemaal niet	Een beetje	Nogal	Veel	Extreem
0	1	2	3	4

57. Uitvallen van haren

Helemaal niet	Een beetje	Nogal	Veel	Extreem
0	1	2	3	4

Complicaties/gevolgen

Heeft u complicaties ondervonden *ten gevolge van de bijwerkingen* gedurende de afgelopen 2 weken?

Bijvoorbeeld:

- o Gewichtstoename of –verlies
- o Vallen, met/zonder botbreuk
- o Flauwvallen
- o (Bijna) ongeval in het verkeer met een voertuig
- o (Huis)artsbezoek
- o Ziekenhuisopname
- o Het niet meer kunnen uitvoeren van bepaalde activiteiten, zoals
  ……………………………………………………………………………………………
  ……………………………………………………………………………………………
  ……………………………………………………………………………………………
- o Overig…………………………………………………………………………………
  ……………………………………………………………………………………………
- o Geen
