# Supplementary material for: Psychometric Properties of the Scale for Subjective Somatic and Cognitive Complaints of Psychotropic Medication Adult‐Aged‐Spectrum (SCOPA)
Source: Hum Psychopharmacol. 2025 Jul 9;40(4):e70009. doi: 10.1002/hup.70009 (PMC12240232; doi:10.1002/hup.70009)
Supplement: Supplementary file 2 — Supporting Information S2 [file HUP-40-e70009-s003.pdf]

Side-effects questionnaire « Scale for Subjective Somatic and Cognitive Complaints of Psychotropic Medication Adult-Aged-Spectrum (SCOPA) »*

Date: ....

Name: .... Male / Female*

Date of birth: ....

Part A

Height (cm)	
Weight (kg)	

Current medication

	Name of medication	Dosage (mg)	Number of tablets per day	For what reason are you taking this medicine?	Starting date (approximately)
1					
2					
3					
4					
5					
6					
7					
8					
9					
10					
11					
12					
13					
14					

Part B

Treatment compliance
How would you judge your treatment compliance?

- Good: you faithfully take your medication in the prescribed manner and at the prescribed times and you rarely or never forget it.
- Moderate: you usually take your medication in the prescribed way and at the prescribed times, but you also regularly (unintentionally) forget it.
- Poor: you usually do not take your medication in the prescribed manner and at the prescribed times.
- You do not take any medication at all that has been prescribed by the doctor.
- You are not prescribed any medication.

** Tick what is applicable to you*

Current complaints
This is about the side-effects or complaints that were present, or have changed in *the last 2 weeks*.

Please circle the most appropriate number (0 = not at all bothered, 4 = very much bothered).

Nervous system

1. Difficulty falling asleep

Not at all	A bit	Somewhat	A lot	Extremely
0	1	2	3	4

2. Problems sleeping through

Not at all	A bit	Somewhat	A lot	Extremely
0	1	2	3	4

3. Forgetfulness: short-term memory problems

Not at all	A bit	Somewhat	A lot	Extremely
0	1	2	3	4

4. Forgetfulness: long-term memory problems

Not at all	A bit	Somewhat	A lot	Extremely
0	1	2	3	4

5. Confusion: Confusion about time, places, persons or events

Not at all	A bit	Somewhat	A lot	Extremely
0	1	2	3	4

6. Difficulty concentrating (for example, while reading, watching television or during conversations)

Not at all	A bit	Somewhat	A lot	Extremely
0	1	2	3	4

7. Headaches

Not at all	A bit	Somewhat	A lot	Extremely
0	1	2	3	4

8. Tense or restless feeling in the body

Not at all	A bit	Somewhat	A lot	Extremely
0	1	2	3	4

9. Irritable and/or aggressive

Not at all	A bit	Somewhat	A lot	Extremely
0	1	2	3	4

10. Overly excited and/or cheerful mood

Not at all	A bit	Somewhat	A lot	Extremely
0	1	2	3	4

11. Depressed mood

Not at all	A bit	Somewhat	A lot	Extremely
0	1	2	3	4

12. Epileptic insult

Yes No

<u>Suicidality</u>

13. (Increased) feelings of wanting to harm yourself

Not at all	A bit	Somewhat	A lot	Extremely
0	1	2	3	4

14. (Increased) thoughts about death

Not at all	A bit	Somewhat	A lot	Extremely
0	1	2	3	4

15. In the past two weeks, have you attempted to end your life?

Yes No

<u>Cardiovascular system</u>

16. Tight feeling of the chest

Not at all	A bit	Somewhat	A lot	Extremely
0	1	2	3	4

17. Heart palpitations

Not at all	A bit	Somewhat	A lot	Extremely
0	1	2	3	4

18. Shortness of breath on slight exertion

Not at all	A bit	Somewhat	A lot	Extremely
0	1	2	3	4

19. Swollen feet or ankles

Not at all	A bit	Somewhat	A lot	Extremely
0	1	2	3	4

20. Badly healing wounds

Not at all	A bit	Somewhat	A lot	Extremely
0	1	2	3	4

21. Easier or longer bleeding from wounds

Not at all	A bit	Somewhat	A lot	Extremely
0	1	2	3	4

Musculoskeletal system

22. Dizziness

Not at all	A bit	Somewhat	A lot	Extremely
0	1	2	3	4

23. Tingling sensation (in arms, legs, hands, feet, etc.)

Not at all	A bit	Somewhat	A lot	Extremely
0	1	2	3	4

24. Decreased muscle strength (for example, in arms or legs)

Not at all	A bit	Somewhat	A lot	Extremely
0	1	2	3	4

25. Feeling of stiffness in muscles (for example, in arms or legs)

Not at all	A bit	Somewhat	A lot	Extremely
0	1	2	3	4

26. Muscle ache

Not at all	A bit	Somewhat	A lot	Extremely
0	1	2	3	4

27. Sensation of uncontrollable movement of muscles (eyes, lips, neck, arms legs, hands, feet)

Not at all	A bit	Somewhat	A lot	Extremely
0	1	2	3	4

28. Restless legs at night

Not at all	A bit	Somewhat	A lot	Extremely
0	1	2	3	4

29. Urge to walk: the feeling that you have to keep walking or that it is difficult for you to sit still

Not at all	A bit	Somewhat	A lot	Extremely
0	1	2	3	4

Gastrointestinal system

30. Increased appetite

Not at all	A bit	Somewhat	A lot	Extremely
0	1	2	3	4

31. Decreased appetite

Not at all	A bit	Somewhat	A lot	Extremely
0	1	2	3	4

32. Nausea

Not at all	A bit	Somewhat	A lot	Extremely
0	1	2	3	4

33. Difficulty making stools (bowel constipation)

Not at all	A bit	Somewhat	A lot	Extremely
0	1	2	3	4

34. Diarrhoea

Not at all	A bit	Somewhat	A lot	Extremely
0	1	2	3	4

35. Excessive thirst, drinking a lot

Not at all	A bit	Somewhat	A lot	Extremely
0	1	2	3	4

36. Dry mouth

Not at all	A bit	Somewhat	A lot	Extremely
0	1	2	3	4

37. Salivation

Not at all	A bit	Somewhat	A lot	Extremely
0	1	2	3	4

<u>Urinary and reproductive system</u>

38. Breast Formation

Not at all	A bit	Somewhat	A lot	Extremely
0	1	2	3	4

39. Frequent urination

Not at all	A bit	Somewhat	A lot	Extremely
0	1	2	3	4

40. Pain during urination

Not at all	A bit	Somewhat	A lot	Extremely
0	1	2	3	4

41. Difficulty urinating (for example, poor flow, dripping)

Not at all	A bit	Somewhat	A lot	Extremely
0	1	2	3	4

42. Increased sexual desire

Not at all	A bit	Somewhat	A lot	Extremely
0	1	2	3	4

43. Decreased sexual desire

Not at all	A bit	Somewhat	A lot	Extremely
0	1	2	3	4

44. Pain during sexual contact

Not at all	A bit	Somewhat	A lot	Extremely
0	1	2	3	4

45. Inability to ejaculate

Not at all	A bit	Somewhat	A lot	Extremely
0	1	2	3	4

46. For men: inability to get an erection. *Women should skip this question.*

Not at all	A bit	Somewhat	A lot	Extremely
0	1	2	3	4

<u>Perception</u>

47. Hearing impairment

Not at all	A bit	Somewhat	A lot	Extremely
0	1	2	3	4

48. Beep or ringing sound in one or both ears

Not at all	A bit	Somewhat	A lot	Extremely
0	1	2	3	4

49. Blurred vision

Not at all	A bit	Somewhat	A lot	Extremely
0	1	2	3	4

50. Double vision

Not at all	A bit	Somewhat	A lot	Extremely
0	1	2	3	4

51. Loss of voice

Not at all	A bit	Somewhat	A lot	Extremely
0	1	2	3	4

52. Changed taste

Not at all	A bit	Somewhat	A lot	Extremely
0	1	2	3	4

53. Hearing, seeing, smelling, feeling or tasting things that other people do not experience

Not at all	A bit	Somewhat	A lot	Extremely
0	1	2	3	4

Integumentary system.

54. Itching

Not at all	A bit	Somewhat	A lot	Extremely
0	1	2	3	4

55. Skin rash

Not at all	A bit	Somewhat	A lot	Extremely
0	1	2	3	4

56. Sweating

Not at all	A bit	Somewhat	A lot	Extremely
0	1	2	3	4

57. Hair loss

Not at all	A bit	Somewhat	A lot	Extremely
0	1	2	3	4

<u>Complications/consequences</u>

Have you experienced any complications *due to the side-effects* during the past 2 weeks? For example:

- o Weight gain or loss
- o Falling, with/without fracture
- o Fainting
- o (Near) traffic accident with a vehicle
- o General practitioner visit
- o Hospitalisation
- o Inability to perform certain activities, such as………
- o Other……
- o None

**This side-effect questionnaire has been freely translated from Dutch towards English. No official APA translation guidelines were used.*
