# Supplementary material for: Psychometric Properties of the Scale for Subjective Somatic and Cognitive Complaints of Psychotropic Medication Adult‐Aged‐Spectrum (SCOPA)
Source: Hum Psychopharmacol. 2025 Jul 9;40(4):e70009. doi: 10.1002/hup.70009 (PMC12240232; doi:10.1002/hup.70009)
Supplement: Supplementary file 3 — Supporting Information S3 [file HUP-40-e70009-s001.docx]

**Appendix 2: legends**

Table 1: this table provides an overview of demographic and clinical characteristics of the in-and outpatient sample, the general population sample and for adults and older adults separately. Sample sizes (n) are shown with percentages (%) or standard deviations (SD).

Table 2: this table provides an overview of different subscales, number of items per scale and the internal consistencies measured with Crohnbach’s alpha (Ca) and the average inter-item correlations (AIC).

Table 3: this table provides an overview of the means and standard deviations (SD) of the in- and outpatient sample and general population sample on the SCOP-A’s scales.

Table 4: this figure contains the rotated component matrix for EFA extraction Method: Principal Component Analysis. Rotation Method: Promax with Kaiser Normalization, three iterations

Table 5: this table provides the prevalence of complications across the total sample, and the in- and outpatient and general population subsample separately. Expressed in frequencies (n) and percentages (%).
